# Mindfulness-Informed Guided Imagery to Target Physical Activity: A Mixed Method Feasibility and Acceptability Pilot Study

**DOI:** 10.3389/fpsyg.2021.742989

**Published:** 2021-12-17

**Authors:** Alexis D. Mitchell, Laura E. Martin, Austin S. Baldwin, Sara M. Levens

**Affiliations:** ^1^Department of Psychological Science, University of North Carolina at Charlotte, Charlotte, NC, United States; ^2^Department of Population Health, University of Kansas Medical Center, Kansas City, KS, United States; ^3^Department of Psychology, Southern Methodist University, Dallas, TX, United States

**Keywords:** guided imagery, physical activity, reward, episodic future thinking, behavior change, mindfulness-informed

## Abstract

Physical activity offers substantial mind-body health benefits and reduced mortality, yet many individuals are chronically underactive. Physical activity interventions may benefit from integrative approaches that join components of mindfulness and neurobiological models of behavior. Mindfulness increases one’s awareness of cognitions and physical sensations to potentially facilitate self-regulation, while neurobiological models such as the dual system model of health behavior offer guidance on improving physical activity intervention targets. This 2-phase study includes an initial development process to create brief (∼4 min) mindfulness informed guided imagery audio files that target distinct cognitive and affective processes to promote physical activity. In the second phase, participants completed a 2-week pilot intervention study to gather qualitative and quantitative data on intervention feasibility and acceptability. Participants endorsed the mindfulness informed guided imagery as easy to use, enjoyable and helpful. Over a 2-week intervention period participants reported positive shifts in behavior change, physical activity enjoyment, increased mindfulness during physical activity, and increased physical exercise self-efficacy and satisfaction. Interview data revealed that participants increased their frequency of physical activity and tended to experience positive affect during physical activity, engaged in future oriented thinking and were able to view physical activity in a more positive light. Findings support the feasibility and acceptability of an integrative online mindfulness informed guided imagery intervention to promote physical activity enjoyment and engagement. This study extends health behavior change intervention research and provides supporting evidence for a flexible and tailorable online mindfulness-based intervention.

## Introduction

Physical activity has been shown to exhibit a wide range of physical health benefits, from improvements in mood (Rebar et al., 2015) to reduced risk for all-cause mortality (Lear et al., 2017). Yet, recent data indicate that 77% of adults 18 and over do not meet federal physical activity guidelines (National Center for Health Statistics, 2018) of engaging in at least 150 min of moderate-intensity aerobic physical activity per week (U.S. Department of Health and Human Services, 2018). Although evidence supports implementation of physical activity interventions for improving individual and population level health (Gardner et al., 2016), there is significant heterogeneity among findings and a lack of clarity regarding what intervention techniques, or what psychological correlates to behavior change, are most effective or involved in influencing physical activity behaviors and attitudes (Howlett et al., 2019). Consequently, it is critical to develop integrative physical activity interventions that incorporate both psychological approaches and neurobiological models to influence physical activity engagement. The present study builds on theoretical and empirical health behavior change research (Hofmann et al., 2008; Schuman-Olivier et al., 2020), existing online and mobile-based physical activity interventions (Bort-Roig et al., 2014; Jahangiry et al., 2017) and online mindfulness based interventions (Toivonen et al., 2017; Cavanagh et al., 2018) to design and test the feasibility and acceptability of a novel, mindfulness-informed guided imagery intervention that targets physical activity related cognitions and emotions to increase physical activity enjoyment and engagement.

Mindfulness, conceptualized as a nonjudgmental stance toward present moment experiences (Kabat-Zinn, 2005), may serve as a mechanism through which positive behavior change can be initiated and maintained to improve health and reduce disease risk (Sagui-Henson et al., 2018; Sala et al., 2020, 2021). In the domain of physical activity, mindfulness has been shown to facilitate the maintenance of physical activity (Ulmer et al., 2010) and promote physical activity satisfaction (Tsafou et al., 2017). Mindfulness is purported to influence health behaviors like physical activity through improved attention regulation (Holzel et al., 2011; Vago and David, 2012), the adoption of a *non-evaluative* stance to the present moment (Baer, 2003), and an increased willingness to experience and accept “negative” sensations (Arch and Craske, 2006). Collectively these processes interact to enhance self-regulation (Holzel et al., 2011) which may increase one’s capacity to maintain goal-directed physical activity behavior in the face of physical discomfort (e.g., labored breathing) and self-limiting judgements (e.g., “I can never be healthy/fit/active”). Mindfulness is a trainable skill (Teasdale et al., 2000; Hayes et al., 2012) and transdiagnostic technique (Baer, 2003; Garland et al., 2014) that can address various maladaptive cognitive, affective, and behavioral patterns associated with mental and physical health concerns and support improvements in stress (Grossman et al., 2004), anxiety (Manzoni et al., 2008), and pain (Chiesa and Serretti, 2011). Mindfulness-informed interventions have also been identified as having wide reaching population level benefits for health behavior change via primary care (Gawande et al., 2019) and workplace settings (Horan and Taylor, 2018), as well as through online-delivery formats for improving health outcomes in the public (Spijkerman et al., 2016). Interventions that incorporate mindfulness therefore have the potential to make meaningful improvement in low engagement health behaviors such as physical activity.

Health behavior research has also identified neurobiological models (Hofmann et al., 2008) that offer guidance on improving physical activity intervention targets. The National Institutes of Health Science of Behavior Change (SOBC) working group (Nielsen et al., 2018) calls for health behavior change interventions to target underlying neurobiological mechanisms that influence behavior, such as specific neural pathways and associated cognitive-emotional, or reflective-affective processes (Hofmann et al., 2008). One prominent health related neurobiological model is the dual-systems model of health behavior (Lieberman, 2007; Hofmann et al., 2008; Wiers et al., 2007). The dual-systems model proposes that health behavior is driven by interactions between the *regulatory*, cognitively effortful (i.e., future-orientation, self-regulated behaviors) and *reward-laden*, habitual (i.e., present-orientation, impulsivity) neural networks in the brain. This framework purports that reasoned actions and self-control involved in healthful behaviors, such as physical activity, may be at conflict with automatic, impulsive affective and behavioral associations. For instance, for physical activity to occur, an individual’s goal and intent to be physically active (regulatory system) would need to override the pleasurable impulse to remain sedentary (reward system), which often does not occur. The interplay between regulation and reward that defines the dual-systems model has been implicated in a range of health behavior interventions, including reducing alcohol (Hamilton et al., 2020) and sugar consumption (Hagger et al., 2017), as well as increasing physical activity levels (Maher and Conroy, 2016; Strobach et al., 2020; Phipps et al., 2021).

According to the dual systems model, health behavior interventions should target either the reward system, the regulatory system, or both. The reward system is targeted via emotional mechanisms, such as positive affect and the facilitation of positive reappraisal of negative stimuli, which has been shown to influence physical activity behavior (Rhodes and Kates, 2009). Positive affect is strongly associated with physical activity motivations (Ekkekakis et al., 2013) and implementing positive reappraisal during physical activity has been shown to improve performance and reduce negative affect during physical activity (Giles et al., 2018). Further, positive associations with physical activity, such as experiencing satisfaction, influence the maintenance of physical activity (Rothman, 2000; Rothman et al., 2011; Baldwin et al., 2013), with satisfaction experienced as little as 2 weeks after initiating regular physical activity predicting physical activity maintenance (Fleig et al., 2011). The dual system model also posits that physical activity behaviors will become habitual when an individual increasingly relies on implicit associations in the reward system (Smith and DeCoster, 2000; Hagger, 2020) as opposed to the regulatory system, suggesting that interventions should target increasing positive associations with physical activity to increase physical activity engagement.

Activation of the regulatory system, on the other hand, involves increased self-regulatory ability and executive functions, such as goal setting and planning (Metcalfe and Mischel, 1999; Hofmann et al., 2008). Physical activity interventions have traditionally targeted the regulatory system through examination of self-regulatory processes like planning and goal-setting (Rhodes and Pfaeffli, 2010). However, many regulatory based physical activity interventions fall prey to the intention behavior gap, in which participants don’t follow through from intention to behavior (Fife-Schaw et al., 2007; Rhodes and Dickau, 2012). Accordingly, health behavior scientists have explored the use of episodic future thinking to target the regulatory system (Dassen et al., 2016; O’Donnell et al., 2017; Sze et al., 2017). Episodic future thinking is a form of prospection that involves directing thoughts to a specific autobiographical experience that may happen in the future (Atance and O’Neill, 2001). Episodic future thinking targets the regulatory system, as executive functioning (e.g., working memory) and attentional processes are required to mentally visualize a subjective future event (Schacter et al., 2017). Episodic future thinking also assists with behavioral regulation by reducing preferences for immediate compared to delayed rewards (Peters and Büchel, 2010; Cheng et al., 2012; Lin and Epstein, 2014). The ability to form internal representations of the future and to abstain from short-term, unhelpful impulses, such as remaining sedentary, is necessary to facilitate goal-directed actions (Bandura, 2001; Barkley, 2001), suggesting that interventions that use physical activity-oriented episodic future thinking may facilitate regulation to increase physical activity engagement.

While mindfulness, positive affect, and episodic future thinking are separate constructs that each have the potential to increase physical activity engagement, one approach that can integrate mindfulness and dual-system reward and regulatory content, is guided mental imagery visualization. Guided mental imagery visualization is regarded as a relaxation-based practice (Luberto et al., 2020) involving multisensory processes in the brain to create vivid mental images of specific places, events, or behaviors, such as themselves engaging in physical activity (Martin and Hall, 1995; Renner et al., 2019). Guided imagery is theorized to influence behavior change and self-regulation by strengthening the link between cognitions and goal-directed actions (Renner et al., 2019) and can be used in a range of contexts to increase motivation toward exercise behavior (Andersson and Moss, 2011; Giacobbi et al., 2014). For example, a brief guided imagery audio intervention that focused on future thinking in relation to physical activity was effective in increasing exercise motivation and frequency of physical activity in a sample of sedentary adults (Andersson and Moss, 2011), illustrating the capacity of guided imagery for delivering episodic future thinking intervention content. Further, guided imagery delivered before or during physical activity is shown to amplify physical activity enjoyment and satisfaction (Stanley and Cumming, 2010), highlighting its capacity to target the reward system outlined in the dual-system model.

Guided imagery is often conceptualized as a relaxation strategy, distinct from mindfulness (Luberto et al., 2020). Importantly, these two mind-body practices have similar qualities and engage similar regulatory processes. For instance, research demonstrates that mindfulness informed (Morton et al., 2020) and guided imagery-based interventions (Bigham et al., 2014) have the potential to reduce stress and improve physiological functioning. Research also shows that multimodal mind-body interventions that integrate both mindfulness and relaxation practices such as guided imagery demonstrate feasibility and efficacy for improving health outcomes (Stagl et al., 2015; Kraemer et al., 2016; Vranceanu et al., 2016). Mindful body scan is an example of a mindfulness informed practice used to increase awareness of bodily sensations that can be implemented through guided mental imagery (Hamilton et al., 2013; Creswell, 2017; Schuman-Olivier et al., 2020). Additionally, mindfulness and relaxation-based techniques are shown to have a similar effect of facilitating cognitive distancing (e.g., taking a step back from negative thoughts and beliefs) (Feldman et al., 2010; Lancaster et al., 2016). Moreover, the present study seeks to pilot test a novel intervention that integrates mindfulness practices, such as mindful breathing, a non-judgmental stance, and attentional awareness with guided imagery, future episodic thinking and positive affect to target reward and regulatory processes in the brain to increase physical activity enjoyment and engagement, an approach that has not yet been tested.

The combination of mindfulness-informed and guided imagery approaches to target reward and regulation processes has the potential to strengthen the underlying neural connections that link physical activity and reward to increase the likelihood of physical activity engagement (Hofmann et al., 2008). Further, the combination of mindfulness-informed and guided imagery approaches to target reward and regulation processes addresses the need for integrative physical activity interventions that incorporate both psychological approaches and neurobiological models to influence physical activity engagement. There is, however, significant heterogeneity between mindfulness-informed and relaxation-based intervention formats (i.e., self-paced, instructional) and deliveries (i.e., audio, classroom/lab setting, online accessibility), making it difficult to extend feasibility and acceptability from one intervention to another. To address critical issues with intervention access inequity (Onken et al., 2014), scalable and accessible interventions are needed (Bedford, 2012) making feasibility and acceptability work in this area particularly important. An integrative intervention that can be delivered remotely and flexibly via a low-cost mainstream online format (e.g., audio file) has the potential to influence physical activity behavior at a large scale.

Accordingly, the present study aimed to test the feasibility and acceptability of a newly developed brief, online mindfulness-informed guided imagery intervention grounded in the dual-system health behavior model and that targets physical activity enjoyment and engagement. This study involved two distinct phases, an intervention development phase and a pilot test phase. Intervention development involved development of four distinct guided imagery scripts that incorporated physical activity-oriented prompts related to goal-setting and episodic future thinking, positive affect and reappraisal, and mindfulness. An iterative intervention development process was employed in which subject matter experts and a panel of underactive community members were asked to provide feedback on intervention content and delivery. The final approved scripts were used to create the intervention audio recordings to be delivered online to a sample of underactive adults.

To obtain feasibility and accessibility data, we employed a mixed-methods approach that collected both self-report and qualitative data from participants on their experiences listening to the guided imagery immediately prior to physical activity. We also implemented a 2 × 2 factorial experimental approach to test the feasibility of conducting a study with different mindfulness-informed guided imagery scripts, in which participants were randomly assigned to one of four mindfulness-informed guided imagery conditions that differentially targeted the reward and regulatory systems proposed in the dual-system model: (1) episodic future thinking, (2) positive affect and reappraisal, (3) combined episodic future thinking and positive affect condition, and (4) control condition with neutral prompts. Findings from the present intervention content and delivery may inform future studies to further improve acceptability, feasibility, and outcomes.

## Materials and Methods

### Phase I: Guided Imagery Development

Phase I of the present study consisted of an iterative intervention development process to create the mindfulness-informed guided imagery content. First, drafts of intervention content were designed based on previously developed guided imagery related to healthy eating (Levens et al., 2019). Four guided imagery scripts were drafted such that each script included mindfulness-based intervention content that specifically targets physical activity enjoyment and engagement: (1) episodic future thinking, (2) positive affect and reappraisal, (3) combined positive affect and reappraisal and episodic future thinking, or (4) a guided imagery control (see Final Intervention Content section for descriptions of the four conditions). Components of mindfulness utilized in the guided imagery scripts included: mindful awareness of the breath, physical sensations of movement, emotional awareness, and linguistic presentation that encourages non-evaluation of one’s physical activity goals, actions, and sensations. For instance, listeners were guided to refrain from using evaluative labels with their experience of physical activity and to consider their goals in relation to their actions in the present moment. All four guided imagery scripts begin with a brief guided breathing exercise and end with bringing awareness to the present moment and one’s emotional state.

#### Professional Advisory Panels

Once initial guided imagery scripts were drafted, feedback was solicited from a professional advisory panel. Members of the professional advisory panel (*N* = 3) volunteered their time to assist with the project. Professional advisors included two doctoral level university researchers in physical activity, affect, cognition, and behavior change, and one master’s level exercise physiologist with extensive applied experience in physical activity intervention implementation in a healthcare setting. Guided imagery scripts were subsequently edited and adapted according to expert feedback. Edits made to intervention content included: (a) shortening the audio files to approximately an average of 3 min and 30 s (with the exception of the combined guided imagery condition, which was shortened as much as possible to ensure content was covered and participants had adequate pauses to follow the guided imagery’s instructions), (b) adding phrases related to increased physical capabilities (e.g., “Perhaps you are doing activities now that you could not before… perhaps these activities take less effort now that you are more active”), (c) addition of explicit instructions for guided breathing at the beginning of each guided imagery, (d) inclusion of word suggestions such as “stronger” and “accomplished” (e.g., “imagine that on this journey you are being physically active… moving your body… accomplishing goals, and becoming stronger, more focused, and healthier”), (e) recommendations to increase the reward value of physical activity (e.g., physical sensations are a sign of one’s body being challenged in a positive way that leads to increases in strength and energy), and (f) inclusion of language that positions the listener’s daily physical activity effort, without evaluation, in the context of their larger goals and health (e.g., “Attach no evaluations to your journey… it is your own;”). Guided imagery scripts were also sent to a Community Advisory Panel for review after the first round of feedback from the professional advisory panel was incorporated.

#### Community Advisory Panel

Following the first round of Professional advisory panel feedback and subsequent adjustments, the scripts and audio files of each guided imagery were edited and then sent to a community advisory panel (*N* = 6) for feedback. The community advisory panel included 3 female and 3 male individuals with an average age of 31.5 years (*SD* = 10.67; range = 21–50), and mostly white (*n* = 5), with one reviewer identifying as Latino. Community reviewer feedback was collected via an electronic survey. Community members provided their impressions of the intervention content via Likert-scale and open-ended questions in a Qualtrics survey. The first round of Community Advisory panel feedback was reviewed and evaluated for appropriateness and congruence with expert feedback, after which the scripts were edited, rerecorded, and then sent to the Professional Advisory panel review for a second round of review. Community Panel members provided specific feedback for refining the scripts. Two participants suggested that the combined condition be simplified and lengthened (e.g., include longer pauses) as it was (1) “too much information” and (2) “easy to lose track while listening.” To address this feedback statements included in the combined condition were streamlined, reordered and longer pauses were added between statements to improve flow. Two participants noted the need for greater inflection in the recorder’s voice to increase the energetic tone of the recording to align with exercise engagement. Each guided imagery condition was re-recorded with a slightly more energetic inflection in the recorder’s voice. Approved guided imagery content from the Professional Advisory panel was then sent back to the Community Advisory panel for a second round of feedback. Both the Professional Advisory and Community Advisory panels reached agreement and saturation (had no additional suggestions to provide) in the second round of review resulting in the finalized guided imagery scripts and audio files.

### Final Intervention Content

Our iterative intervention development yielded four guided imagery scripts/audio files that differentially target physical activity enjoyment divided into four experimental conditions: (1) episodic future thinking, (2) positive affect and reappraisal, (3) combined positive reappraisal and episodic future thinking condition, and (4) a neutral control condition. All intervention scripts/audio files were designed to include elements of mindfulness. Each intervention condition began with a brief mindful breathing exercise, and included content that (1) encouraged a non-evaluative stance toward physical activity (i.e., “Attach no evaluations to your [physical activity] journey…it is your own.”), (2) a focus on mind-body sensory engagement (e.g., imagery aimed to engage senses as if they were there in the moment they were imagining), and (3) guided participants to redirect their focus to their mental image (i.e., “It’s ok if your mind wanders just return to this vision of yourself”) or the present moment (i.e., “Bring your attention back to the present. Awaken your muscles so you can continue on your journey and ready them for activity”).

The *episodic future thinking* condition (audio length: 3 min 26 s) targets non-evaluative future thinking and visualization of physical activity benefits for future self, goal achievement, and the impact of effort on overall health. In this guided imagery condition, in accordance with episodic future thinking techniques, listeners are prompted to visualize a future ideal version of themselves in 6 months accomplishing a physical activity goal. Sample statements include: “take a moment to create a picture in your mind of a future ideal version of yourself – a future version of yourself who is healthy, strong and embodies what you strive for…” and “Now, 6 months have passed on your journey. Imagine that you have accomplished one of your physical activity goals.” See Supplementary Materials for full script.

The *positive affect and reappraisal* condition (audio length: 3 min 56 s) aimed to increase likelihood of experiencing positive affect associated with physical activity (e.g., enjoyment and satisfaction) through prompting positive introspection about the benefits of physical activity and feelings experienced after physical activity is completed (e.g., accomplished, satisfied). Example statements include: “…the increase in sensations can be a good thing… your body is responding to the challenge you are giving it and becoming stronger, healthier, energized…” as well as “imagine that you’ve finished your activity and you feel satisfied, accomplished, confident, and energized… let this feeling build.” See Supplementary Materials for full script.

The *episodic future thinking and positive affect and reappraisal condition* [combined guided imagery] (audio length: 5 min 35 s) integrates the two aforementioned conditions to assess the impact of both episodic future thinking and positive affect and reappraisal on the experience of physical activity. All intervention content from the episodic future thinking and positive affect and reappraisal only conditions are included in the combined condition, and content that is common between both conditions is stated once. See Supplementary Materials for full script.

Finally, the guided imagery *control condition* (audio length: 3 min 9 s) focuses on the physical actions of a neutral, routine activity that occurred in the last week. The control condition was designed to promote mindful practice and include the delivery components of the intervention conditions (guided mindful breathing, prompted mindfully vivid recall of an event, and normalization of mind wandering and language to redirect listener’s attention back to the present as the guided imagery concludes) without the target intervention content of episodic future thinking and positive affect and reappraisal (i.e., reward/positive affect focus, future thinking, and link to broader health was absent in the control condition). For instance, the control condition includes the following statement: “Imagine a moment where you were doing a simple activity, an activity that you do every day.” See Supplementary Materials for full script.

### Intervention Mode of Delivery

The intervention content was designed to be delivered as an online audio file that could be accessed directly via participants’ smart phones through their email or as a saved audio file. Participants were informed they could download the guided imagery audio file directly to their personal device to ensure access even without internet. Audio files were recorded using Audacity audio recording software. Participants received a scheduled email each day in the morning of the 2-week intervention period. This daily email contained a link to the audio file for their assigned intervention condition as well as an online survey taken after engaging in physical activity to assess physical activity and intervention access experience. Participants only had access to the audio file for the condition to which they were assigned at the Part 1 session. Each participant was instructed to listen to their assigned audio file prior to engaging in physical activity each time they engage in physical activity over a 2-week period. Given that the sample consisted of underactive adults with varying health statuses, we instructed participants to listen to the guided imagery audio file before physical activity a minimum of three times (see Procedures for more detail).

### Phase II: Feasibility Pilot Study Methods

#### Participants

Thirty-one participants were recruited in a metropolitan area in the southeast United States through a 4-year state university research listserv announcement available to the campus community. The feasibility pilot study was advertised as an opportunity to engage in a remote intervention targeting physical activity enjoyment. Interested participants completed a pre-screen eligibility survey embedded in the listserv announcement.

#### Eligibility

Study inclusion criteria required that participants were over the age of 18, fluent in English, did not meet recommended physical activity guidelines and were considered underactive at time of study participation (i.e., engage in less than 60 min of moderate or vigorous physical activity a week), had an internet accessible device to play the mindful guided imagery audio file, and the capacity to engage in physical activity safely (access to safe areas to engage in physical activity). Physical activity levels were determined by the International Physical Activity Questionnaire (Craig et al., 2003). Exclusion criteria included having a medical diagnosis in which a physician advises against engaging in physical activity and engaging in more than 60 min of moderate or vigorous physical activity at time of study participation. This pre-screen eligibility survey was programmed to determine participant eligibility at the point of completion. Eligible participants were subsequently contacted by the research team for scheduling.

#### Design

As this is a pilot study to test the feasibility and acceptability of an integrative mindfulness-informed guided imagery intervention in anticipation of conducting larger scale clinical trials, we employed a 2 × 2 factorial design commonly used to isolate main and interactive effects of condition. While our pilot sample size of 31 participants does not support any 2 (future episodic thinking present vs. absent) by 2 (positive affect and reappraisal present vs. absent) factorial analyses or between-group comparisons, we sought to use the design to maximize feasibility and acceptability inferences from data collection. This approach also allowed us to test randomization and experimental procedures.

#### Randomization

Participants were randomized to one of four intervention conditions, which represented the four distinct guided imagery audio files. To achieve randomization, each condition was assigned a number and a random order generator determined the allocation assignment sequence. The randomly generated allocation sequence was listed in a spreadsheet and participants were assigned their condition sequentially at their intake session before they were provided training on accessing and using the guided imagery audio file. Participants were blinded to their condition assignment and were unaware that there were multiple intervention conditions. The experimenter facilitating the participant’s intake and follow-up session adhered to the preset, random assignment order and was knowledgeable of condition assignment to provide participants with their assigned intervention audio file.

#### Procedure

To limit in-person transmission of the coronavirus and test the feasibility of remote intervention enrollment, all study protocols were implemented virtually and remotely. All study procedures were approved by the university’s Institutional Review Board. Participants were informed that the study comprised three parts: a virtual intake session (Baseline – Part 1), a 2-week intervention use time period (Intervention Use – Part 2), and a virtual outtake session approximately 14 days after Part 1 (Follow-up – Part 3). At the virtual intake session, participants were lead through consent and enrollment by the experimenter on a video chat platform. Participants completed a battery of questionnaires (see Table 1) and were sent their initial intervention use email which included the guided imagery audio file (mp4). During the 2-week intervention period, participants received a daily email that provided (1) online access to their assigned guided imagery audio file and (2) an online survey to be completed immediately following each physical activity session. The daily online survey assessed their physical activity and intervention use experience. To provide attainable goals for a sample of underactive adults, participants were asked to engage in physical activity and listen to the guided imagery beforehand for a *minimum* of 3 times. Participants were instructed they could exceed this number if they desired and that if they did, each session of exercise should begin with listening to their assigned audio file and end with completing the online survey. Participants were instructed that if they did not engage in physical activity that day, they should complete the survey at night before bed.

**TABLE 1:** SPIRIT Figure: Schedule of enrolment, intervention, and assessments.

	STUDY PERIOD							
	Recruitment	Baseline	Guided Imagery Use					Follow-up
TIMEPOINT	*−t_1_*	*t*_1_	*T*_2_	−	−	−	*T*_13_	*T*_14_
**ENROLMENT:**								
Eligibility screen	X							
Informed consent		X						
Allocation		X						

**INTERVENTIONS:**								
*Episodic Future Thinking*		X	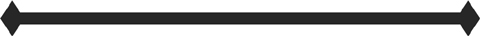					
*Positive Affect and Reappraisal*		X	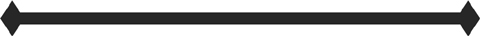					
*Combined: Episodic future Thinking and* *Positive Affect and Reappraisal*		X	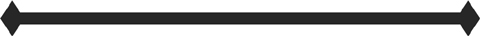					
*Control*		X	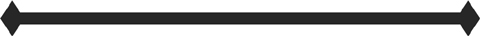					

**ASSESSMENTS:**								
*Demographics*		X						
*Mental Health*		X						
*Physical Health*		X						
*Enjoyment with PA*			X	X	X	X	X	
*Physical Activity Behaviors (duration, intensity, type)*			X	X	X	X	X	
*PA Satisfaction*		X						X
*Mindfulness during PA*		X						X
*PA Self-Efficacy*		X						X
*Stages of PA Behavior Change*		X						X
*Feasibility Self-Report*			X	X	X	X	X	X
*Acceptability Self-Report*			X	X	X	X	X	X
*Semi-structured feedback interview*								X

*This is a SPIRIT figure that highlights what measures were delivered at each time point for the pilot test phase. PA, physical activity.*

For each bout of exercise, we asked participants to engage in at least 15 min of physical activity and encouraged them to reach a level of moderate intensity, such that they could feel slight changes in physical sensations: labored breathing, increased sweat, and increased heart rate. Participants were given leeway to select the intensity, duration, and activity type of their choice. In our instructions we emphasized the need for participants to listen to their body as not to increase risk for injury. We asked each participant to identify safe physical activity options during the interview to confirm their access to safe physical activity options. Participants were not provided any other instructions.

Following the 2-week intervention period, participants returned to the lab for a virtual follow-up session. During this follow-up session, participants completed a battery of questionnaires that included measures assessed at baseline and self-report questions to assess their experience using the intervention. This follow-up session also included a brief audio recorded semi-structured interview in which participants were asked to provide feedback on intervention acceptability. Participants were compensated $40 in electronic gift cards for their time and debriefed about the goal of study of influencing physical activity enjoyment.

#### Measures

##### Demographic Information

Participants self-reported demographic information in the Part 1 baseline survey which included age, sex, gender identity, racial and ethnic identity, educational attainment, occupational status, presence of mental and physical health conditions, and household income. Participants’ physical activity levels were assessed in the eligibility questionnaire using International Physical Activity Questionnaire (Craig et al., 2003).

#### Intervention Use Data

Intervention feasibility and acceptability were assessed through mixed-method self-report and coded interview data. Participants were asked to (1) listen to the provided guided imagery audio file, (2) engage in a physical activity of their choice, and (3) complete the intervention use daily survey immediately following physical activity (the daily survey ended if participants endorsed not listen to the guided imagery that day).

##### Intervention Adherence

We defined intervention engagement as the number of times the participant listened to the guided imagery immediately before physical activity. This was assessed through the daily survey participants were asked to complete following physical activity (or before bed if they did not engage in physical activity that day). Participants were asked whether they listened to the guided imagery and engaged in physical activity afterward. If “yes,” participants were provided additional questions to complete. See Table 1 for measurement assessment throughout the study.

##### Type of Physical Activity

Participants were asked to provide a qualitative description of the physical activity they completed (e.g., “Describe in detail the activity you completed”). Responses from the entire sample were coded by type of physical activity.

##### Physical Activity Intensity

Participants were asked to self-rate the physical activity intensity they engaged in (“Please rate the intensity (degree of increased heart and breathing rate) of your activity based on your own self-rating…”) using the following response options*:* 1 = *Not intense*, 2 = *Slightly intense*, 3 = *Moderately intense*, and *Very intense*). Given that survey submissions varied by participant based on number of intervention uses, responses were averaged for each participant and then again across conditions submissions for an average sample intensity score.

##### Physical Activity Duration

Participants were asked to report the length of their physical activity bout using the following response options: (1) *Less than 10 min* (2) *10 min*, (3) *15 min*, (4) *20–30 min*, (5) *30–45 min*, (6) *45–60 min*, (7) *More than 60 min*. Responses were calculated in same manner as physical activity intensity.

##### Self-Report Feedback During Intervention Use

Participants completed three self-report feasibility and acceptability questions at the end of the daily us survey. Questions targeted enjoyment (“I enjoyed listening to the guided imagery”), ease of use (“I found the guided imagery easy to use”), and whether they found the guided imagery helpful (“I found the guided imagery helpful”). Participants responded using a 5-pt Likert scale (1 = *Strongly disagree*, 5 = *Strongly agree*). As the number of times participants listened to the intervention and engaged in physical activity varied, responses for each self-report question were averaged for each participant, with higher scores indicating positive feasibility and acceptability.

##### Physical Activity Enjoyment

The intervention use daily survey included the Physical Activity Enjoyment Scale (Mullen et al., 2011) to assess physical activity enjoyment. Physical activity enjoyment is regarded as a distinct affective experience derived from physical activity that may influence satisfaction and intrinsic motivation (Rhodes et al., 2009). Participants rated 8 items that assessed how they felt in the moment about the physical activity just completed (e.g., “It’s very gratifying”) using a 7-point Likert scale (1 = *Strongly disagree*, 4 = *Neither agree nor disagree*, 7 = *Strongly agree*). Participant responses were summed by instance and then averaged across the 2-week period for each participant and then for the sample.

#### Intervention Feasibility and Acceptability at Follow-Up

To assess intervention use, feasibility, and acceptability at follow-up, participants were asked to complete both self-report questions related to intervention use, feasibility, and acceptability, as well as a qualitative semi-structured interview designed to gather additional data on feasibility and acceptability (see Table 1).

##### Self-Report Feedback

Self-report questions assessed delivery quality, ease of use of delivery format (i.e., audio file in an electronic survey included in a daily email), whether directions and prompts in the intervention content were clear and easy to follow, applicability of the intervention content to other areas of their life, and likelihood of using the intervention outside the context of the present study. Participants also provided ratings on their overall experience with the intervention content, including: degree of acceptability, satisfaction, whether the individual thought about the intervention content during their physical activity, whether the intervention content made the participant want to increase their physical activity levels, and whether they could see other individuals in their life enjoying and benefiting from the intervention content to increase their physical activity levels. All questions were answered using one of the following 5-point Likert scales: 1 = *Totally disagree* to 5 = *Totally agree*; or 1 = *Extremely dissatisfied* to 5 = *Extremely satisfied*; or 1 = *Not at all acceptable* to 5 = *Extremely acceptable*.

##### Interview Feedback

Open-ended interview questions pertained to the participant’s initial impressions of the intervention content, barriers and facilitators influencing their use of the guided imagery, most and least helpful components of the intervention content, and feedback relating to the degree to which the intervention content influenced physical activity levels, enjoyment of physical activity, and could be used outside the context of the present study.

#### Outcome Measures

##### Stage of Physical Activity Behavior Change

Current stage of physical activity behavior change was assessed using a previously validated measure (Romain et al., 2012) derived from the Transtheoretical Model of Behavioral Change (Prochaska and DiClemente, 1983). Participants are asked to respond to “yes” or “no” to the following questions which each represent a different stage of health behavior change, (1) “Do you currently engage in regular physical activity?,” (2) “Do you intend to engage in regular physical activity in the next 6 months?,” (3) “Do you intend to engage in regular physical activity in the next 30 days?” and (4) “Have you been regularly physically active for the past 6 months?”. The scale specifically defines ‘regular physical activity’ as at least 30 min a day for 4 days of the week. Stage calculation is based on participants’ responses on to each of the above items. Stages include: (1) pre-contemplation stage answering ‘no’ to all four items (i.e., individual does not intend to take action and could be unaware of the pros of changing their behavior), (2) contemplation stage answering yes to “Do you intend to engage in regular physical activity in the next 6 months? (i.e., individual is intending to start the behavior but may still be ambivalent toward changing their behavior), (3) preparation stage answering ‘yes’ to “Do you intend to engage in regular physical activity in the next 30 days? (i.e., individual is ready to act and small steps toward behavioral change and likely believe in the positive outcomes of the behavior), (4) action stage answering ‘yes’ to “Do you currently engage in regular physical activity?” and ‘no’ to “Have you been regularly physical activity for the past 6 months?” (i.e., individual has recently changed their behavior and will be more likely to consider ways to further modify their behavior), and (5) maintenance stage: answering yes to “Do you currently engage in regular physical activity?” and “Have you been regularly physically active for the past 6 months?” (i.e., individual has sustained a behavior change and likely works to prevent relapse of negative health behaviors, like remaining sedentary). This measure was included at baseline and follow-up (see Table 1).

##### Physical Activity Self-Efficacy

Perceived self-efficacy has been noted as a driving force for forming intentions to exercise and maintaining the practice over an extended period (Sheeran et al., 2016). The 5-item Physical Exercise Self-Efficacy Scale (Schwarzer and Renner, 2000) was included to assess the degree to which individuals feel certain they can overcome barriers to engaging in physical activity. It is a 5-item questionnaire in which participants are prompted to respond to the following statement, “How certain are you that you could overcome the following barriers…” with a 4-point scale (1 = *Very uncertain*, 4 = *Very certain*). Example items include “even when I am busy” and “even when I am tired.” Item responses are summed to yield a total score, with higher scores indicating higher self-efficacy for physical exercise (score range = 4 = 16). This scale demonstrates good internal consistency (α = 0.88) (Sheeran et al., 2016). This measure was assessed at baseline and follow-up (see Table 1).

##### Mindfulness in Physical Activity

Given that this study was designed to incorporate elements of mindfulness, a measure was included to assess the guided imagery’s potential impact mindful qualities experienced during physical activity. The Mindfulness in Physical Activity (MFPA) (Tsafou et al., 2016) six-item scale was included to measure state mindfulness experienced during physical activity. It is a validated measure that demonstrates good internal consistency (α = 0.84). The questionnaire begins with the statement “When I am doing physical activity” followed by six items (e.g., “I am focused on what I am doing”). Responses are on a 5-point Likert scale (1 = *Totally disagree*) to (5 = *Totally agree*) and are summed. Higher summary scores represent greater levels of mindfulness during physical activity. This measure was included at a baseline and follow-up (see Table 1).

##### Satisfaction With Physical Activity

Satisfaction with physical activity was measured using an eight-item scale developed for a previous study (Tsafou et al., 2017). This scale extends previous one-item assessments of satisfaction (Baldwin et al., 2013) to assess satisfaction with engagement in (i.e., during) and outcomes of physical activity. This measure demonstrates excellent internal consistency (α = 0.90). The scale stem, “When I am doing physical activity,” is followed by items such as “I am satisfied with the results.” Responses are on a 5-point Likert scale (1 = *Totally agree*) to (5 = *Totally disagree*) and summed across items. Higher summary scores indicate greater levels of satisfaction with physical activity. This measure was assessed at baseline, during intervention use, and follow-up (see Table 1).

#### Data Analysis

We conducted both quantitative and qualitative analysis of the data. As this is a pilot study focused on feasibility and acceptability of an online mindfulness-informed guided imagery intervention with insufficient power to conduct factorial analyses or explore effect sizes, our quantitative analyses collapsed across condition and focused on sample wide outcome measures and feasibility and acceptability observations. Descriptive statistics were conducted for all demographic variables, outcome measure data, intervention use responses, and self-report feasibility and acceptability responses. To assess whether data meets acceptability and feasibility guidelines, we followed implementation science recommendations stipulating that the cut-off for determining an intervention as acceptable is an average rating of 4.0 or higher on 5-point scales with questions like “How acceptable was your experience with the guided imagery?” (4 = *Acceptable*, 5 = *Extremely Acceptable*) and “The guided imagery was easy to use” (4 = *Agree*, 5 = *Strongly Agree*) (Proctor et al., 2011; Weiner et al., 2017). To examine change in outcome measures across the course of the study, we conducted paired *t*-tests or Wilcoxon signed-rank tests comparing baseline and follow-up summary scores for Stage of Behavioral Change, Exercise Self Efficacy, Mindfulness in Physical Activity, and Satisfaction with Physical Activity. Wilcoxon signed-rank tests were conducted to change for any outcome variables with either a baseline or follow assessment with a kurtosis or skew value greater than 1.0.

A thematic coding procedure was performed with all qualitative interview data collected from the semi-structured interviews. First, transcripts for each interview were checked alongside audio recordings for accuracy by research assistants. Second, participant interview responses were then coded by a quantitative methods trained senior graduate-level researcher based on themes demonstrated in the following interview question categories: Initial impressions of the guided imagery, delivery method, facilitators and barriers, most and least liked/helpful components of the guided imagery, influence on physical activity behaviors and experience of physical activity, generalizability and improvements to the guided imagery, and use beyond present study. An inductive coding and theme development process (Terry et al., 2017) was conducted to determine the dominant themes within each category for each condition. Themes were then compared across conditions for similarity and frequency of occurrence to determine broader full sample level themes.

## Results

Results are presented in four sections. The first section presents condition assignment and demographic data (see Table 2). The second section presents intervention use and physical activity statistics (see Table 3) as well as self-report feedback assessed during intervention use (see Table 4). The third section includes self-report data (see Table 5) and thematic coded interview data collected at follow-up (see Table 6 for full sample and individual condition themes). The final section presents baseline and follow-up comparisons of outcome measures (e.g., for Stage of Physical Activity Behavior Change, Physical Exercise Self Efficacy, Mindfulness in Physical Activity, Satisfaction with Physical Activity; see Table 7).

**TABLE 2:** Demographics.

	Full Sample	EFT	Positive Affect	Combined	Control
				
Demographic Variable	*N* = 31	*n* = 8	*n* = 8	*n* = 8	*n* = 7
				
Age M (SD)	29.16 (10.66)	28.25 (9.53)	25 (6.95)	31.38 (12.12)	32.43 (13.84)
				
	*N* (%)	*N*	*N*	*N*	*N*
**Gender**					
Female	19 (61%)	4	5	4	6
Male	11 (35%)	4	3	4	−
Non-binary	1 (3%)	−	−	−	1
**Race/Ethnicity**					
White	15 (48%)	4	3	4	4
Black	5 (16%)	1	1	1	2
Latinx	4 (12.9%)	1	1	1	1
Asian	4 (12.9%)	2	1	1	−
Middle Eastern	2 (6.5%)	−	2	−	−
Multi Racial	1 (3%)	−	−	1	−

*N = 31. EFT, episodic future thinking condition: n = 8, positive affect (positive affect and reappraisal condition): n = 8, combined (combined episodic future thinking and positive affect and reappraisal condition): n = 8, control condition: n = 7.*

**TABLE 3:** Physical activity intervention use data.

	Full Sample	EFT	Positive Affect	Combined	Control
			
	M (SD)	M (SD)	M (SD)	M (SD)	M (SD)
Number of GI listens before PA engagement	5.35 (2.99)	4.75 (2.19)	4.0 (1.77)	6.50 (3.82)	6.57 (3.46)
PA duration	4.45 (1.29)	5.03 (1.04)	4.13 (1.18)	4.26 (1.55)	4.40 (1.12)
PA intensity	2.67 (0.60)	2.74 (0.76)	2.56 (0.98)	2.44 (0.93)	2.76 (0.61)

*N = 31. GI: guided imagery; PA, physical activity; EFT, episodic future thinking: n = 8, positive affect (positive affect and reappraisal): n = 8, combined condition (combined future thinking and positive affect and reappraisal): n = 8, control condition: n = 7. Physical activity duration (1–7 scale): 1 = Less than 10 min, 2 = 10 min, 3 = 15 min, 4 = 20–30 min, 5 = 30–45 min, 6 = 45–60 min, 7 = More than 60 min. Physical activity intensity (1–4 scale): 1 = Not intense, 2 = Slightly intense, 3 = Moderately intense, 4 = Very intense.*

**TABLE 4:** Self-report feedback during intervention use.

	Full Sample	EFT	Positive Affect	Combined	Control
	
Feedback	M (SD)	M (SD)	M (SD)	M (SD)	M (SD)
I enjoyed listening to the GI.	4.03 (0.72)	3.92 (0.43)	3.87 (1.01)	4.34 (0.75)	3.89 (0.53)
I found the GI easy to use	4.47 (0.67)	4.41 (0.60)	4.38 (0.55)	4.55 (0.74)	4.58 (0.50)
I found the GI helpful.	4.00 (0.82)	3.89 (0.46)	3.81 (0.99)	4.36 (0.72)	3.82 (0.91)
PA Enjoyment Scale	5.14 (0.79)	5.07 (0.96)	5.29 (1.00)	5.11 (0.53)	5.01 (0.69)

*N = 31. GI, guided imagery; PA, physical activity; EFT, episodic future thinking: n = 8, positive affect (positive affect and reappraisal): n = 8, combined condition (combined future thinking and positive affect and reappraisal): n = 8, control condition: n = 7. These feedback questions were presented with a 5-pt Likert scale (1 = Strongly disagree, 5 = Strongly agree). Number of survey entries: full sample = 164 (EFT = 38; positive affect = 32; combined = 52; control = 42). Participant responses for physical activity enjoyment were first summed and then averaged across all entries over the 2-week intervention period. Number of total GI uses before physical activity: Full sample = 164 (EFT = 38; positive affect = 32; combined = 52; control = 42). Physical Activity Enjoyment Scale (Craig et al., 2003): 7-point Likert scale (1 = Strongly disagree, 2 = Disagree, 3 = Somewhat disagree, 4 = Neither agree nor disagree, 5 = Somewhat agree, 5 = Agree, 7 = Strongly agree).*

**TABLE 5:** Self-report feasibility and acceptability at follow-up.

	Full Sample	EFT	Positive Affect	Combined	Control
	
Feedback Area	M (SD)	M (SD)	M (SD)	M (SD)	M (SD)
GI was acceptable	4.65 (0.55)	4.62 (0.52)	4.50 (0.76)	4.62 (0.52)	4.86 (0.38)
Satisfied with GI	4.26 (0.82)	4.13 (0.99)	4.25 (1.04)	4.50 (0.54)	4.14 (0.69)
GI was easy to use	4.90 (0.30)	5.00 (0.00)	4.88 (0.35)	4.88 (0.35)	4.86 (0.38)
Instructions in GI were clear and easy to follow	4.90 (0.30)	4.88 (0.35)	4.88 (0.35)	4.88 (0.35)	5.00 (0.00)
Liked having access to GI on phone	4.03 (1.08)	4.25 (0.89)	3.38 (1.30)	3.75 (1.04)	4.86 (0.38)
Listening to GI was a pleasant experience	4.52 (0.68)	4.25 (0.89)	4.63 (0.52)	4.63 (0.52)	4.57 (0.78)
Thought about GI during PA	3.77 (1.09)	4.00 (1.07)	3.25 (1.28)	4.25 (0.46)	3.57 (1.27)
Would use again for PA	4.06 (1.03)	4.38 (0.74)	3.50 (1.41)	4.50 (0.54)	3.86 (1.07)
GI changed view of PA	3.58 (1.03)	3.63 (1.06)	3.63 (1.41)	3.62 (0.74)	3.43 (0.98)
Motivated to increase PA	4.06 (0.89)	4.13 (0.64)	3.50 (1.31)	4.38 (0.52)	4.29 (0.76)
Useful in other aspects of life	4.10 (1.04)	3.75 (1.04)	3.87 (1.46)	4.38 (0.74)	4.43 (0.79)
Other people would benefit to increase PA	4.19 (0.98)	4.13 (1.13)	3.88 (1.36)	4.50 (0.54)	4.29 (0.76)
Other people would benefit to increase enjoyment	4.06 (0.89)	4.25 (1.06)	3.63 (1.19)	4.25 (0.46)	4.14 (0.69)

*N = 31. GI, guided imagery; EFT, episodic future thinking: n = 8, positive affect (positive affect and reappraisal): n = 8, combined condition (combined future thinking and positive affect and reappraisal): n = 8, control condition: n = 7. Participants responded on a 5-pt Likert scale (1 = Totally disagree, 5 = Totally agree).*

**TABLE 6:** Qualitative feedback on each guided imagery from semi-structured feedback interviews.

Qualitative themes	Full Sample	EFT	Positive Affect	Combined	Control
**Initial impressions**	% (N)	% (N)	% (N)	% (N)	% (N)
I1. Increased focus	32% (10)	16% (5)	9.7% (3)	6.5% (2)	−
I2. Increased motivation	32% (10)	6.5% (2)	9.7% (3)	9.7% (3)	6.5% (2)
I3. Relaxing	23% (7)	−	9.7% (3)	−	12.9% (4)
**Delivery method**					
D1. No issues and Easy to use	65% (20)	23% (7)	16% (5)	16% (5)	9.7% (3)
D2. Preference for variety	42% (13)	16% (5)	9.7% (3)	9.7% (3)	6.5% (2)
D3. Quicker access	19% (6)	6.5% (2)	3.2% (1)	6.5% (2)	3.2% (1)
D4. Headphones	9.7% (3)	3.2% (1)	−	−	6.5% (2)
D5. Length is ideal	23% (7)	−	16% (5)	3.2% (1)	3.2% (1)
D6. Length not ideal	12.9% (4)	6.5% (2)	3.2% (1)	−	3.2% (1)
D7. Routine/memorized	16% (5)	6.5% (2)	3.2% (1)	6.5% (2)	−
**Facilitators to use**					
F1. Benefits and change	52% (16)	12.9% (4)	12.9% (4)	12.9% (4)	12.9% (4)
**Barriers to use**					
B1. No barriers	52% (16)	9.7% (3)	12.9% (4)	16% (5)	12.9% (4)
B2. Time and Schedule	19.4% (6)	3.2% (1)	9.7% (3)	−	6.5% (2)
B3. COVID-19 Limitations	6.5% (2)	−	−	6.5% (2)	−
B4. Life stressors	12.9% (4)	3.2% (1)	9.7% (3)	−	−
**Helpful components**					
H1. Voice	35% (11)	6.5% (2)	9.7% (3)	6.5% (2)	12.9% (4)
H2. Increased positive thinking	23% (7)	−	12.9% (4)	9.7% (3)	−
H3. Goal clarification	39% (12)	6.5% (2)	12.9% (4)	19.4% (6)	−
H4. Awareness of future	36% (11)	19.4% (6)	−	16% (5)	−
**Unhelpful components**					
U1. Nothing unhelpful	48% (15)	9.7% (3)	16% (5)	9.7% (3)	12.9% (4)
U2. Pace and tone	36% (11)	12.9% (4)	9.7% (3)	6.5% (2)	6.5% (2)
U3. Imagery content	9.7% (3)	6.5% (2)	−	−	3.2% (1)
**Influence on PA**					
PA1. Positive mindset	90% (28)	19.4% (6)	25.8% (8)	25.8% (8)	19.4% (6)
PA2. Mindfulness	32% (10)	12.9% (4)	3.2% (1)	9.7% (3)	6.5% (2)
PA3. Frequency and Intensity	71% (22)	12.9% (4)	25.8% (8)	16% (5)	25.8% (5)
PA4. Motivated by future	23% (7)	12.9% (4)	−	9.7% (3)	−
PA5. No change PA enjoyment	29% (9)	9.7% (3)	9.7% (3)	6.5% (2)	3.2% (1)
**Generalizability/Improvements**					
G1. Post-PA reflection GI	12.9% (4)	−	6.5% (2)	6.5% (2)	−
G2. Inclusive language	6.5% (2)	−	6.5% (2)	−	−
G3. Voices and languages	9.7% (3)	−	3.2% (1)	3.2% (1)	3.2% (1)
G4. Other people would benefit	26% (8)	9.7% (3)	9.7% (3)	6.5% (2)	−
G5. Change GI format	16% (5)	6.5% (2)	6.5% (2)	3.2% (1)	−
G6. Changes to GI prompts	19% (6)	3.2% (1)	9.7% (3)	−	6.5% (2)
G7. Phone reminders	9.7% (3)	−	6.5% (2)	3.2% (1)	−
G8. Would use outside study	65% (20)	9.7% (3)	19% (6)	26% (8)	9.7% (3)

*Qualitative themes for each category of feedback are presented here. Full sample: N = 31. PA, physical activity; GI: guided imagery; EFT, episodic future thinking: n = 8, positive affect (positive affect and reappraisal): n = 8, combined condition (combined future thinking and positive affect and reappraisal): n = 8, control condition: n = 7. Intervention content was described as a “guided imagery” to participants. Theme descriptions: (I1) increased focus for PA, (I2) more motivated to complete PA, (I3) intervention content as “relaxing,” “soothing,” and/or “calming.” (D1) no issues and easy to use and access, (D2) would like different audio files to rotate, (D3) would like quicker access through a mobile phone app, (D4) audio quality better with headphones, (D5) length of the audio file was ideal, (D6) length of the audio file was not ideal: would like to be longer or shorter, (D6) became routine or memorized after 2–3 listens. (F1) Began feeling hopeful about change and had noticed the positive benefits of listening to GI. (B1) Denied barriers to use and access, (B2) time and/or busy schedule, (B3) limitations due to COVID-19 (e.g., gym closures), (B4) life stressors distracted them. (H1) Endorsed voice as “soothing,” “encouraging,” and that it “sounded like a friend,” (H2) more positive thinking about themselves and their PA, (H3) opportunity to set intentions and clarify PA related goals, (H4) able to deepen their connection between present moment and future outcomes. (U1) Nothing was unhelpful, (U2) pace and tone described as inappropriate: instructions were either too slow or too quick, too many discernible pauses in the audio file, and/or GI was “too calming,” (U3) did not like the content of the GI, such as the future focus or routine activity. (PA1) Increased positive affect and shift to a positive mindset about PA, (PA2) increased mindfulness, attention, and engagement during PA, (PA3) increased frequency and intensity of PA, (PA4) felt more motivated during their PA from thinking of future outcomes, (PA5) GI did not increase or change their level of enjoyment experienced during PA. (G1) Desire to have a reflection based GI to listen to after PA, (G2) maintain inclusive language that can apply to the broader population, (G3) different voices and languages (e.g., Spanish), (G4) people in their life would also benefit from the GI and find it easy to use, (G5) noted that the GI could be paired with exercise instructions, workout videos, and/or embedded within a group/exercise class, (G6) need for specific changes to the intervention content: planning statement (e.g., when, where, etc., will you complete physical activity), (G7) mid-day push notification reminders, (G8) would use the GI in everyday life.*

**TABLE 7:** Means and standard deviations of outcome measures for full sample and conditions.

	T1 – Baseline	T2 – Follow-up	
Outcome Measure	M (SD)	M (SD)	Change Difference
**PA Stage of Change**	2.94 (0.68)	3.54 (1.25)	0.6
Future thinking	3.00 (1.07)	3.13 (1.46)	0.13
Positive affect	2.63 (0.74)	3.63 (1.30)	1.00
Combined	3.13 (0.35)	4.00 (1.00)	0.87
Control	3.00 (0.00)	3.43 (1.27)	0.43
**PA Self-Efficacy**	10.39 (3.53)	12.19 (3.30)	1.80
Future thinking	9.50 (3.21)	12.50 (4.99)	3.00
Positive affect	9.13 (4.02)	11.13 (2.85)	2.00
Combined	12.13 (2.36)	12.88 (2.48)	0.75
Control	10.86 (3.39)	12.29 (2.56)	1.43
**Mindfulness in PA**	21.94 (4.18)	23.90 (3.64)	1.96
Future thinking	22.89 (4.22)	25.00 (5.18)	2.11
Positive affect	20.25 (5.55)	22.13 (2.36)	1.88
Combined	22.63 (2.97)	24.50 (3.70)	1.87
Control	22.00 (3.83)	24.00 (2.45)	2.00
**PA Satisfaction**	23.52 (3.97)	25.29 (2.64)	1.77
Future thinking	24.38 (4.17)	27.00 (2.14)	2.62
Positive affect	20.63 (4.27)	24.38 (3.82)	3.75
Combined	25.75 (3.58)	25.12 (1.96)	−0.63
Control	23.29 (1.80)	24.57 (1.51)	1.28

*N = 31. PA, physical activity. Stage of Behavior Change Scale: 1 = pre-contemplation stage (i.e., individual does not intend to take action and could be unaware of the pros of changing their behavior), 2 = contemplation stage (i.e., intending to start the behavior but may still be ambivalent toward changing their behavior), 3 = preparation stage (i.e., ready to act and small steps toward behavioral change and likely believe in the positive outcomes of the behavior), 4 = action stage (i.e., has recently changed their behavior and will be more likely to consider ways to further modify their behavior), 5 = maintenance stage (i.e., has sustained a behavior change and likely works to prevent relapse of negative health behaviors, like remaining sedentary).*

### Descriptives and Demographics

Participants (*N* = 31) consisted of underactive individuals (i.e., individuals who engage in less than 60 min of physical activity at time of study participation) who ranged from absent to lower levels of physical activity. At time of study participation, 48% (*n* = 15) reported zero engagement in any moderate or vigorous physical activity, 16% (*n* = 5) participants reported engaging in 0–45 min of moderate or vigorous physical activity in the previous week, and 36% (*n* = 11) participants reported engaging in 45–60 min of moderate-vigorous physical activity. See Table 2 for participant demographics (age, gender, race/ethnicity) across the full sample and by condition.

### Intervention Use Data

Intervention use data (physical activity intensity, duration, self-report feasibility and acceptability, and physical activity enjoyment) were averaged across each participants’ number of daily survey submissions due to variation in number of reported intervention uses and subsequent instances of physical activity. Full sample and condition averages for intervention use and physical activity statistics are presented in Table 3, and self-report intervention use feedback is presented in Table 4.

During the 2-week intervention period and across the four conditions, the entire sample used the intervention audio files before physical activity a total of 164 times, with a range of 3 to 12 uses. Average use of the guided imagery across all conditions was 5.35 (*SD* = 2.99). Participants reported engaging in physical activity that included an element of cardio or aerobics (e.g., walking, jogging, using a cardio machine like a stepper or elliptical machine, playing a sport outside such as soccer) paired with another activity like stretching the most frequently (85 instances), followed by just cardio (59 instances), strength training (e.g., body-weight exercises, lifting weights) and cardio combined (37 instances), use of a video or virtual fitness class (26 instances), or yoga, Pilates, and stretching (25 instances).

In regard to the intensity level of activities, participants reported engaging in moderately intense activities most frequently (76 instances), followed by slightly intense (52 instances), very intense (21 instances), and not intense (16 instances) activities. Average physical intensity ratings endorsed across all conditions were between slightly intense and moderately intense. Participants reported an average physical activity duration between 20–30 min and 30–45 min of physical activity (see Table 3). Participants reported engaging in physical activity for 20–30 min most frequently (72 instances), followed by 30–45 min (29 instances), 45–60 min (28 instances), 15 min (18 instances), greater than 60 min (9 instances), and finally 10 min or less (8 instances).

Descriptives of self-report questions asked during the intervention use daily survey are presented in Table 4. On a 5-point Likert style scale (4 = *Agree*, 5 = *Strongly Agree*), participants rated the guided imagery as enjoyable to listen to (*M* = 4.08, *SD* = 0.72), easy to use (*M* = 4.47, *SD* = 0.82), and helpful (*M* = 4.00, *SD* = 0.82). In terms of enjoyment experienced during physical activity (7-point Likert scale, 7 = *Strongly agree*), participants indicated that the guided imagery on average influenced their degree of enjoyment experienced during physical activity (*M* = 5.14, SD = 0.79).

### Intervention Feasibility and Acceptability at Follow-Up

Self-report feasibility and acceptability responses assessed at follow-up are summarized in Table 5. Overall, participants endorsed high satisfaction with the intervention, *M* = 4.26, *SD* = 0.82 (Likert scale, 1 = *Extremely dissatisfied* to 5 = *Extremely satisfied*) and rated it as acceptable, *M* = 4.65, *SD* = 0.82 (Likert scale, 1 = *Not at all acceptable* to 5 = *Extremely acceptable*). Participants also endorsed the guided imagery files as clear and easy to follow (*M* = 4.90, *SD* = 0.30) and easy to use (*M* = 4.90, *SD* = 0.30; 5-point Likert scale (1 = *Totally disagree* to 5 = *Totally agree*).

Responses to the semi-structured interview were also coded for intervention use, feasibility, and acceptability themes (see Table 6). Responses to one question that related to a different question or category were included with the related category or theme.

#### Initial Impressions

Thirty-two percent of all participants described feeling more focused on physical activity after listening to the guided imagery, and more motivated to complete physical activity after listening (*n* = 9), with 7 of these 9 participants noting they were surprised by how much the imagery influenced their motivation for physical activity. Thirty-two percent of all participants also reported that their impression was that the guided imagery motivated physical activity. Lastly, 23% of the sample described the guided imagery as relaxing, calming, and/or soothing.

#### Delivery Method Feedback

Participants across all four intervention conditions volunteered the following themes regarding the guided imagery delivery method: 65% of the sample described having zero issues with the delivery method and/or noted the delivery method as easy to use and access, which included being able to access the imagery through their personal/cellular device. Nineteen percent of participants noted a desire for quicker access to the guided imagery through a mobile application and 10% noted that the guided imagery was best listened to with headphones. Additionally, 29% of the sample described that the length of the guided imagery as ideal, with a small subset noting that the length of the imagery needed to be lengthened or shortened. Sixteen percent reported that that the imagery became “routine” and/or “memorized” after 2–3 uses. Lastly, 48% of participants reported that the guided imagery should be delivered with “different” or “multiple” versions that, for example, could be tailored to “how you’re feeling that day” and “different ability levels” to reduce boredom with imagery and increase likelihood of routine use.

#### Facilitators and Barriers

Participants were asked to describe the barriers or challenges they confronted with listening to the guided imagery before engaging in physical activity, as well as the facilitators, or reasons why, they chose to utilize the guided imagery. In the context of barriers, 52% (*n* = 16) of participants endorsed zero specific barriers or challenges to using the guided imagery for physical activity. Of the participants that endorsed barriers, 19% noted time and busy schedules as a barrier, 13% noted life stressors as distracting, and two participants described COVID-19 limitations as a barrier (e.g., not being able to work-out at a gym). Regarding facilitators, 53% of participants described a desire to use the guided imagery because of feeling hopeful about change (e.g., increase physical activity, feel more joy during physical activity) or noticing positive benefits of using the guided imagery during study. For instance, one participant in the combined condition described feeling motivated to use the guided imagery because “Everything I have tried in the past to motivate myself has not worked.” Additionally, participants in the combined condition described using the guided imagery for the positive reinforcement offered through the guided imagery (*n* = 4) as well as to have “accountability” and “motivation” when feeling tired before or during physical activity (*n* = 2).

#### Most Helpful and Least Helpful Components

Of the full sample, 35% endorsed that the voice of the guided imagery was one of the most helpful components, in that it was “soothing,” (*n* = 7), “encouraging,” (*n* = 3), and “sounded like a friend” (*n* = 1). Several participants described that they found thinking more positively about their exercise and themselves as most helpful. Additionally, 39% of participants liked the opportunity for intention setting and goal clarification and 36% found it helpful to deepen their connection between themselves and their actions in the present with themselves in the future. For example, a participant in the combined condition described that the guided imagery helped them identify the “larger purpose of physical activity” in benefiting their future. Further, one participant in the future thinking condition stated, “it was an opportunity to check in with my emotions and connect this with doing physical activity” and that the guided imagery included “inclusive language” that allowed the participant to create a visual image of “my own version of what healthier looks like.”

Themes identified in relation to unhelpful aspects of the guided imagery included that some of the guided imagery instructions were slow to progress, that the guided imagery was “too calming” before physical activity, the specific focus of guided imagery did not appeal to them and some noted discernible pauses in the guided imagery that caused a degree of distraction. Finally, 48% of the sample noted that there were no specific aspects of the guided imagery that they found unhelpful.

#### Influence on Physical Activity Behaviors and Experience of Physical Activity

Across the full sample, 90% of participants noted an increase in positive emotions (e.g., pride, achievement, satisfaction, enjoyment) and experienced a shift to a more positive mindset (e.g., empowered, change in thinking about physical activity) before and during physical activity that they attributed to the guided imagery. For instance, one participant in the future thinking condition described feeling “more empowered and less distraught” about physical activity, whereas another participant in the positive affect and reappraisal condition described that they experienced increased enjoyment in a brief period, stating they felt the guided imagery could “rewire your brain.” Thirty-two percent of participants also described experiencing increased mindfulness, attention, and engagement during physical activity. When asked about how the guided imagery influenced their physical activity engagement, participants qualitatively described the guided imagery as beneficial for increasing physical activity: 71% of participants reported an improvement in physical activity behaviors, including an increase in frequency and intensity. Additionally, 23% of participants expressed feeling more motivated for physical activity due to realizing how their actions and behaviors were going to benefit their future. Lastly, 10 participants specifically noted that the guided imagery *did not* increase their degree of enjoyment experienced during physical activity.

#### Generalizability and Improvements to Guided Imagery

Participants were asked to provide feedback on what would increase the generalizability and applicability to other individuals and suggested improvements. The following themes were described in relation to generalizability: two participants suggested creating a reflection based guided imagery to listen to *after* physical activity to reaffirm physical activity cognitions and behaviors and link them back to the guided imagery, another two participants suggested that it would be important to maintain inclusive language, with one of these participants suggesting that we continue to ensure that that content does not focus on weight and can be tailored. One participant suggested the imagery be adapted for different hearing abilities. Three participants also suggested that different voices (e.g., dialects) and languages would increase generalizability. Of the full sample, 26% noted that other individuals they knew (e.g., friends and parents) would find the guided imagery motivational and/or easy to use for physical activity.

Lastly, of the suggested changes participants provided, two themes were identified across the sample: (1) pair guided imagery with a workout video, instructions, or embed within group exercise format and (2) to initiate specific changes to the intervention content to include components of the guided imagery present in conditions other than the condition the participant was randomized to, such as adding a planning statement (e.g., when, where, etc., will you complete physical activity), ensure that the imagery includes statements about physical activity goals, that the guided imagery focused on goals, and in the context of challenging/busy schedules and lives, one participant in the positive affect condition suggested the inclusion of a statement that encourages listeners to “ignore outside stressors.” One participant in the future thinking condition suggested adding a “sympathetic” statement to the future thinking condition that acknowledges how difficult physical activity can be. Lastly, 10% of participants reported that mid-day phone reminders to listen to the guided imagery and complete physical activity would be a helpful improvement to increase likelihood of regular use.

#### Use This Guided Imagery Outside Context of Study

Participants were asked to provide feedback on whether they would use the guided imagery outside the context of the present study. Of the full sample, 65 % described that they would use the guided imagery in the future in some capacity, with 39% of this subset of the sample noting they would utilize the guided imagery specifically for physical activity (e.g., to increase intensity, frequency, motivation, etc.). The other 26% of the participants stated that they would use the guided imagery for contexts not directly related to physical activity, such as for academic assignments or projects (e.g., with “big writing projects”), to maintain a “growth mindset” toward physical activity, to be “more optimistic,” and to change eating patterns.

### Outcome Measures

To examine whether any key outcome measures changed across the course of the study, we conducted descriptive statistics (see Table 7) and paired *t*-tests or Wilcoxon signed-rank tests comparing baseline and follow-up summary scores for intervention outcome variables. Each outcome variable measurement was evaluated for kurtosis and skewness to test for normal distribution of the data. Stage of Physical Activity Behavior Change baseline scores, kurtosis of 5.87 (*SE* = 0.82), and Satisfaction with Physical Activity follow-up scores, skewness of – 1.19 (*SE* = 0.42) and kurtosis of 3.90 (*SE* = 0.82), were non-normally distributed and compared via Wilcoxon signed-rank tests; all other variables showed normal distribution and were analyzed via paired *t*-tests. The Wilcoxon signed-rank test revealed that Stage of Physical Activity Behavior Change scores increased significantly from baseline to follow-up, *Z* = –2.63, *p* = 0.009. On average, participants shifted (*M* = 0.6, *SD* = 1.0) to a *higher* stage of change. In addition, the full sample averaged to be within the ‘preparation stage’ range of physical activity behavior change at follow-up (*M* = 3.54, *SD* = 1.25), which indicates that most of the sample endorsed that they intended to engage in regular physical activity in the 30 days after the study ended. A Wilcoxon signed-ranks indicated that Satisfaction with Physical Activity significantly increased from baseline to follow-up, *Z* = –2.71, *p* = 0.007. In addition, Physical Exercise Self-Efficacy, *t* (30) = 2.73, *p* = 0.011 and Mindfulness in Physical Activity *t* (30) = 2.98, *p* = 0.006 all increased significantly from baseline to follow-up assessment (see Table 7 for means across Time 1 and Time 2).

## Discussion

The overarching goal of this study was to develop, and pilot test an integrated intervention that combines mindfulness with guided imagery and targets underlying mechanisms involved in health behavior engagement, specifically reward and regulatory processes (Hofmann et al., 2008). Our integrative design approach incorporates mindfulness, positive affect, and episodic future thinking into a guided imagery to promote physical activity enjoyment and engagement. This study is the first, to our knowledge, to include both mindfulness and dual-system reward and regulation content into an online accessible guided imagery intervention designed to promote physical activity. Given that this intervention approach has not been empirically tested before, we aimed to explore the feasibility and acceptability of our novel intervention in a sample of physically underactive adults.

Findings consistently support the feasibility and acceptability of the intervention content and its delivery. According to our benchmark cutoff of above 4 on a 5-point scale (Proctor et al., 2011), full sample level findings indicate that participants found the guided imagery to be acceptable, satisfactory, easy to use and access, and that included instructions were clear and easy to follow, with an average acceptability rating of 4.65 (*SD* = 0.55) for the study. We applied this benchmark set by intervention implementation research to the self-report feedback questions included in this study. Questions included cover similar concepts to those included in the validated Acceptability Intervention Measure (AIM) and Feasibility of Intervention Measure (FIM) (Weiner et al., 2017). In addition, reported physical activity behavior supports our feasibility findings – participants used the guided imagery before physical activity an average of 5.35 times, on average 2.5 *more* uses over the 2-week intervention period than required (study required minimum of 3 uses). In addition, participants engaged in a range of physical activities at slight to moderate intensity with an average exercise bout between 20 and 45 min in length. Participants’ self-report feedback also indicated that they tended to enjoy listening to the guided imagery and found it to be helpful. Given that this sample was comprised of underactive adults, these feasibility findings are promising. Integrative interventions such as this, delivered in a simple and straightforward way as online audio recording, have the potential to help individuals reach the recommend 150 min a week of moderate-intensity aerobic physical activity guideline (U.S. Department of Health and Human Services, 2018).

Participants also consistently rated the delivery method positively. A little over 50% of participants described having no barriers or challenges to using the guided imagery prior to physical activity, with a small portion of the sample endorsing environmental barriers to use, including busy schedules, life stressors, and COVID-related limitations (e.g., gym closure). Over 60% of the sample qualitatively described the delivery method as easy to use and access. Participants liked having access to the guided imagery on their phone, with a small subset of participants describing during the interview that they prefer *quicker* access to the guided imagery recording through a mobile application on their phone, or the pairing of the guided imagery with mid-day phone reminder notifications.

Approximately 30% of the sample reported the length of the guided imagery recording as appropriate. Interestingly, although the combined guided imagery condition was the longest, length did not appear to have a strong influence on intervention acceptability or overall perceptions for participants in this condition. Also, a subset of participants (less than 20%) noted that the guided imagery content became routine and/or memorized after approximately 3 uses. The quick assimilation of the intervention content as routine after just a few uses is promising as it suggests the formation of positive habitual associations that could implicitly cue physical activity. It is also possible that mindfulness components of the intervention help facilitate the retainment of intervention content. Mindfulness training has been shown to support free-recall and enhance episodic memory recall in a recognition memory task (Brown et al., 2016). The quick assimilation of the intervention content, however, may also suggest that individuals could become bored or disinterested in the guided imagery content due to lack of variability. Two participants noted that the guided imagery may be “too calming” before physical activity, and about one-third of participants reported that they would be more likely to use the guided imagery if it could be delivered through different variations, perhaps based on physical activity level or other individual characteristics or preferences. The potential to tailor the intervention content and provide participants with multiple guided imagery recording options (i.e., access to audio files from all 4 intervention arms) is a promising feasibility and acceptability future direction.

In regard to increasing physical activity engagement, participants agreed that the intervention content motivated them to increase their physical activity. About 70% of the sample expressed during their interview that they noticed an increase in their physical activity engagement, including increase in physical activity frequency, duration, and intensity. In addition, over 25% of the sample stated that the guided imagery increased their motivation and readiness for physical activity, with some participants noting surprise at their own increase in motivation. These findings suggest that the intervention may be acceptable even for individuals who may be initially hesitant to try a new approach like mindfulness-informed guided imagery. In terms of physical activity outcome measures, we found an overall positive shift in stage of behavior change and physical exercise self-efficacy across the entire sample. This finding suggests that the intervention may have increased physical activity intentions from a contemplation to preparation stage. Participants also reported an increase in physical activity self-efficacy which may have increased perception of one’s ability to engage in physical activity. Participants provided a range of reasons for listening to the guided imagery before physical activity, including feeling hopeful about experiencing a change in their physical activity level, noticing some immediate benefits derived from using the guided imagery (e.g., increased motivation), benefiting from positive reinforcement for physical activity, and using the guided imagery for motivation for physical activity when tired. The collective reporting of increases in motivation during and following the intervention use period also supports the overall feasibility and acceptability of the intervention content and delivery.

As this is the first online mindfulness-informed physical activity intervention that also incorporates content to target reward and regulatory pathways, our goal was to test sample wide feasibility and acceptability, not the differential impact of the mindful, reward and regulatory intervention components. Nevertheless, some of our findings offer proof of concept that our intervention components are meeting their intended targets. In terms of mindfulness, participants experienced a significant increase in state mindfulness during physical activity across all intervention conditions. While evidence suggests higher trait mindfulness is beneficial for physical activity promotion (Chatzisarantis and Hagger, 2007; Sala et al., 2020), the ability to be mindful during physical activity may confer additional unique benefits to physical activity engagement. Mindfulness techniques have been shown to influence physical activity attitudes as physical activity is performed (Salmon et al., 2010), and when used before physical activity, mindfulness increases physical activity endurance by increasing awareness of one’s bodily sensations, facilitating the adjustment of effort (Chatzisarantis and Hagger, 2007). About one-third of participants explicitly noted experiencing increased mindfulness and attention (e.g., focused on movements and effort, not distracted by thoughts or stressors) during physical activity. Additionally, a third of participants noted that the guided imagery increased their ability to focus on physical activity. A sharpened focus and attention before beginning physical activity may have encouraged greater mindfulness during physical activity. These findings suggest that the mindfulness-informed content appears to have promoted mindful practice during physical activity, however, additional research with a larger sample is needed to test intervention efficacy, and so we draw this conclusion cautiously.

In regard to targeting the reward and regulatory pathways outlined in the dual-system model, our findings also provide proof of concept that positive associations with physical activity are occurring. Participants reported that they experienced enjoyment during physical activity when using the intervention audio files, and participants rated the intervention content as enjoyable and pleasant to listen to. Almost the entire sample (90%) noted an increase in positive emotions (e.g., pride, achievement, satisfaction, enjoyment) and experienced a more positive mindset (e.g., empowered, change in thinking about physical activity) before and during physical activity after listening to the intervention content. Finally, we found that self-reported satisfaction with physical activity increased from baseline to follow-up. These findings are encouraging as enjoyment is an important motivator of physical activity maintenance (Williams et al., 2008, 2012) and satisfaction has been noted as an important facilitator for underactive individuals are initiating activity (Baldwin et al., 2013). Further, in combination with the reported increases in exercise motivation and shift in stage of behavioral change, the present findings support the influence of positive physical activity valuations on physical activity behavior change (Rhodes and Kates, 2009; Rhodes et al., 2019) and experimental intervention approaches targeting affective, reward-laden processes (Strobach et al., 2020; Phipps et al., 2021). Of note, however, about 29% of the sample described open-endedly that the intervention content did *not* have a significant impact on how much they enjoyed physical activity. As the majority of participants expressed an increase in positive emotions and positive mindset before and during physical activity as a result of the guided imagery, this pattern of findings suggests that there may be underlying differences in how individuals derive enjoyment from physical activity, an important area for future investigation.

Our intervention also included episodic future thinking guided imagery content (Renner et al., 2019) for targeting regulatory pathways in the brain. The incorporation of episodic future thinking into a mindfulness-informed guided imagery recording to increase goal-orientation for physical activity is novel and feasibility has not been tested. About 40% of the full sample indicated that the intervention content facilitated clarification of their physical activity goals and intentions. In addition, participants in the episodic future thinking condition, and combined condition (which included episodic future thinking content), tended to describe goal-orientation in more detail during their interview. A subset of participants in both the episodic future thinking (6 of 8) and combined (5 of 8) guided imagery conditions stated that the imagery strengthened their mental connection between their present self and their future self and the recognition of larger, long-term benefits of physical activity for improving health. Although this qualitative data is from a small sample and should be interpreted with caution, the findings provide proof of concept that the episodic future thinking content is targeting regulatory processes that encourage delay of gratification of immediate impulses in favor of long-term rewards. In combination with earlier reported findings on increased motivation and greater than anticipated rates of physical activity engagement, the present pattern of findings also supports prior research that episodic future thinking prior to physical activity can increase exercise behaviors over a 2-week period (Andersson and Moss, 2011). As empirical evidence purports that regular physical activity is associated with greater valuation of the future (Daugherty and Brase, 2010; Garza et al., 2013), the present findings also provide proof of concept for the feasibility and acceptability of including episodic future thinking in a mindfulness-informed guided imagery intervention targeting physical activity. Replicating and extending the present finds with a larger sample is an essential next step in confirming the benefits of episodic future thinking on physical activity enjoyment and engagement.

One intriguing finding related to the feasibility and acceptability of our integrative mindfulness based guided imagery intervention is the potential for individual differences in preferences to drive which intervention condition would be most beneficial or liked. For instance, two participants, in the positive affect and reappraisal condition and the control conditions, reported a desire to include a focus on goal setting in the guided imagery. Whereas two participants in the episodic future thinking condition described the future-orientation as unhelpful, and that the 6-month future timeframe did not resonate with them. Supporting the general acceptability of a mindfulness-informed guided imagery approach, participants in the control condition tended to rate the guided imagery as calming and relaxing, with positive feedback regarding ease of use, enjoyment, and physical activity mindfulness all within similar ranges of the target intervention conditions. However, one participant in the control condition found thinking about a routine activity unhelpful. Finally, participants who received the episodic future thinking, positive affect and reappraisal, and control condition guided imagery files each described a need to include components of the guided imagery content present in the *other* conditions to which they were not assigned (i.e., addition of more positive warmth/sympathy to the episodic future thinking condition, and the addition of goal setting to the positive affect and control conditions). This feedback supports our integrative approach as it highlights the need for intervention content that caters to a range of emotional and cognitive processing styles. Yet it also highlights the need for intervention feasibility and acceptability research that incorporates personal preference and needs, a future goal of our research team.

The present study sought to use best practices for increasing the efficacy and effectiveness of health behavior change interventions. The Science of Behavior Change (SOBC) working group at the National Institutes of Health identified the need to design and test integrative, evidence-based approaches to increasing physical activity that expand on decades of health behavior change grounded in theory (Nielsen et al., 2018). The SOBC working group asserts that it is necessary to target and test underlying psychological mechanisms and antecedents in intervention research. Behavior change theories are heterogeneous, but most similarly emphasize the importance of regulatory processes in influencing behavior change (Rebar et al., 2015; Hennessy et al., 2020). For physical activity, however, evidence has increasingly pointed to the influence of positive affect, habitual, and reward laden processes for physical activity initiation and maintenance (Hagger, 2020). Overall, the present study contributes to research and addresses SOBC’s recommendations for improved intervention design and feasibility testing through an experimental approach that targets regulatory and reward-laden constructs potentially involved in physical activity health behavior change.

While the present study has notable strengths of in terms of design and integrative approach, there are several limitations that should be considered when interpreting results. The sample size is too small for conducting analyses that would compare intervention outcome variables between conditions. The sample size is also too small for capturing a broader range of inter-subject variability, or for detecting nuances in intervention preferences that could affect acceptability or efficacy. While we used industry standard benchmarks similar to Acceptability Intervention Measure (AIM) (Weiner et al., 2017), formally including the AIM with its *a priori* acceptability benchmarks would have facilitated incorporating our findings into the broader intervention implementation and acceptability field of research. To further leverage a factorial design, future research with an adequate larger sample size may formally incorporate measures such as the AIM and compare the efficacy of the four conditions on physical activity enjoyment and engagement. In addition, physical activity engagement was assessed via self-report rather than via objective methods such as an ActiGraph. Future research testing intervention efficacy should use objective measures of physical activity engagement such as ActiGraph, smart watch activity trackers, or physiological recordings of physical activity intensity, duration, and exertion. Our sample was also highly educated and limited in diversity, which limits potential generalization to the general population. Additionally, as some participants suggested, our guided imagery were only available in English. Future research should record the intervention content in other languages such as Spanish to examine feasibility and acceptability in marginalized ethnic groups. Relatedly, feasibility and acceptability should be examined in other specialized populations, such as under resourced individuals living with chronic pain (Garland and Howard, 2013) and cancer (Zernicke et al., 2013) who may have physical activity limitations but would also benefit from flexible, highly tailorable approaches to increasing physical activity. Finally, there are individual differences that may influence intervention use that were not examined in this study, such as trait mindfulness (Baer et al., 2008; Kharlas and Frewen, 2016) and specific personality traits (Tang and Braver, 2020) – future research incorporating these psychological traits has the potential to inform intervention feasibility, acceptability, efficacy, and intervention personalization.

Despite these limitations, there are multiple strengths of this feasibility and acceptability pilot study. The study presents a novel, online and remotely available guided imagery intervention that integrates mindfulness content with content targeting reward and regulatory pathways of the dual-system model of health behavior (Hofmann et al., 2008). This study also implemented best practice recommendations by the SOBC working group at the National Institutes of Health – we tested overarching feasibility and acceptability of a mindfulness-informed guided imagery intervention and used an experimental approach (Nielsen et al., 2018) to obtain proof of concept of the mindfulness, positive affect and reappraisal, and episodic future thinking intervention components. In addition, our design prioritized scalability and accessibility – the intervention is brief, easily accessible online and offline (after downloading to a personal device) and can be used at any point in the day to cue physical activity. Remotely available interventions may have the greatest potential for scalability to wider populations and may be particularly important for underactive adults seeking to increase their physical activity (Vandelanotte et al., 2007).

Accordingly, we tested the feasibility and acceptability of our novel physical activity enjoyment and engagement intervention in a sample of *underactive* adults. While our sample size limits generalizability and efficacy testing of our intervention conditions, sample wide findings demonstrate increases in state mindfulness during physical activity, an increased positive mindset toward physical activity, increased focus on goals and intentions for physical activity, and increases in physical activity self-efficacy and satisfaction. Finally, the present study illustrates how an integrative intervention approach can provide access to multiple intervention targets in a mindfulness-informed guided imagery format that could be tailored to meet individual needs and preferences. Findings from this study have the potential to address gaps in physical activity intervention research and expand and clarify how mindfulness is integrated into health behavior interventions. Results also suggest novel areas of future research that examine underlying psychological mechanisms and intervention antecedents to increase efficacy rates for physical activity and other health behavior interventions.

## Data Availability Statement

The raw data supporting the conclusions of this article will be made available by the authors, without undue reservation.

## Ethics Statement

The studies involving human participants were reviewed and approved by the University of North Carolina at Charlotte Institutional Review Board (Protocol # 19-0651). The patients/participants provided their written informed consent to participate in this study.

## Author Contributions

AM and SL designed the study with input from AB and LM. AM and SL applied for human subject IRB approval, drafted and revised all intervention content, and drafted the manuscript. AM collected Expert and Community Advisory panel feedback, oversaw pilot data collection, and conducted thematic analysis coding of the interview data. SL oversaw quantitative data analysis. LM served as a member of the expert advisory panel. LM and AB provided feedback on drafts of the manuscript. All authors contributed to the article and approved the submitted version.

## Conflict of Interest

The authors declare that the research was conducted in the absence of any commercial or financial relationships that could be construed as a potential conflict of interest.

## Publisher’s Note

All claims expressed in this article are solely those of the authors and do not necessarily represent those of their affiliated organizations, or those of the publisher, the editors and the reviewers. Any product that may be evaluated in this article, or claim that may be made by its manufacturer, is not guaranteed or endorsed by the publisher.
