# Supplementary material for: Mindfulness-Informed Guided Imagery to Target Physical Activity: A Mixed Method Feasibility and Acceptability Pilot Study
Source: Front Psychol. 2021 Dec 17;12:742989. doi: 10.3389/fpsyg.2021.742989 (PMC8719519; doi:10.3389/fpsyg.2021.742989)
Supplement: Supplementary file 1 [file Table_1.pdf]

Additional Demographics

Demographic Variable	Full Sample *N = 31*	EFT *n = 8*	Pos Affect *n = 8*	Combined *n = 8*	Control *n = 7*
Income					
≤ $24,999	9 (29%)	2	3	3	1
$25,000 – 75,000	10 (32%)	3	2	1	4
> $75,000	12 (39%)	3	3	4	2
Occupational Status					
Full time student	16 (51%)	5	5	4	2
Employed full-time	12 (38%)	3	1	4	4
Employed part-time	3 (9%)	--	2	--	1
Educational Status					
Some college	15 (48%)	3	4	4	4
College degree	6 (19%)	2	3	--	1
Grad/professional	10 (32%)	3	1	4	2
Mood/anxiety disorder	14 (45%)	4	4	2	3
Physical Health	2 (6.5%)	1	--	1	
High blood pressure					1
Pre-diabetes	3 (9.7%)	2	1	--	--
Obesity	9 (29%)	3	2	2	3
Type 2 Diabetes	1 (3.2%)	--	--	--	1

Note. *N* = 31. Mood/anxiety disorder = participants self-reported 'Yes' or 'No' to a checklist of conditions that have been diagnosed by a medical professional; 5 of the 14 participants endorsed being diagnosed with both an anxiety and a mood disorder. Physical Health = participants self-reported 'Yes' or 'No' to a checklist of conditions that have been diagnosed by a medical professional.
