# Supplementary material for: Mindfulness-Informed Guided Imagery to Target Physical Activity: A Mixed Method Feasibility and Acceptability Pilot Study
Source: Front Psychol. 2021 Dec 17;12:742989. doi: 10.3389/fpsyg.2021.742989 (PMC8719519; doi:10.3389/fpsyg.2021.742989)
Supplement: Supplementary file 2 [file Data_Sheet_1.PDF]

Episodic Future Thinking Condition

Beginning

Begin by getting into a comfortable position

First, take a deep breath in and… exhale fully … continue breathing deeply, releasing any tension in your body as you exhale…. Next, take a slow breath and focus on how your body feels as you breathe…

notice the natural rhythm of your breath, and continue breathing evenly in this way…. (transition)

Prompt Future thinking/Future self

Now, take a moment to create a picture in your mind of a future ideal version of yourself—a future version of yourself who is healthy, strong and embodies what you strive for... Take a moment to think about this future version of yourself that is healthier and fulfilled.

Cue convo w/ experimenter and begin thinking of journey to future, ideal self

Now, as you picture this ideal version of yourself… imagine the journey you take toward your future self… imagine that on this journey you are being physically active…moving your body…accomplishing goals, and becoming stronger, more focused, and healthier…

Future goal thinking

Now, take a moment to think about the physical activity goals you are striving for.

Select a physical activity to do today and imagine yourself doing this physical activity.

Think about how the steps you take today are getting you closer to accomplishing your goals. Today is a part of this journey to becoming this future, ideal version of yourself. Attach no evaluations to your journey…it is your own.

Now, 6 months have passed on your journey. Imagine that you have accomplished one of your physical activity goals.

Where might you be? .. … How do you feel? …Picture who might be around you...

…Perhaps you are doing activities now that you could not before… perhaps these activities take less effort now that you are more active

try to stay with this image of your future self in 6 months … Hold it in your mind.

It’s ok if your mind wanders just return to this vision of yourself, as you carry it with you to today…. *(transition)*

Ending

Now…bring your attention back to the present. Awaken your muscles so you can continue on your journey and ready them for activity…

Positive Affect and Reappraisal Condition.

Beginning

Begin by getting into a comfortable position

First, take a deep breath in and… exhale fully … continue breathing deeply, releasing any tension in your body as you exhale…. Next, take a slow breath and focus on how your body feels as you breathe…

notice the natural rhythm of your breath, and continue breathing evenly in this way…. (transition)

Cues convo w/ experimenter and positive thinking of PA

Now, Create a picture in your mind of a healthy body that is radiating positivity. This body has strong muscles and a vital heartbeat. It feels energized and positive.

Now, imagine yourself being physically active and building energy within your body. Select a physical activity to do today and imagine yourself doing this physical activity. Think of how doing this physical activity will expand your physical capabilities. Think about the positive benefits of this activity for you… how it might enable you to do more than you could before, and make your daily life feel more enjoyable…

Build on this and take a moment to imagine yourself doing this activity as vividly as you can… imagine your body moving…

Reframe physical aspects as challenge

What sensations are you feeling as your body moves? Is your heart beating faster, harder?…Do you feel your muscles contracting?…. as your effort increases, your breathing and physical sensations may intensify. Your body might feel warm…you might feel fatigued…you may want to label this experience as bad, or uncomfortable—recognize that this is an arbitrary label.

…Rather, the increase in sensations can be a good thing… your body is responding to the challenge you are giving it and becoming stronger, healthier, energized…

Attach no evaluations to your physical activity journey. Through your effort you are increasing your strength and physical capabilities, making the next time you exercise easier, and more familiar….

Satisfaction from finishing PA

Now, imagine that you've finished your activity and you feel satisfied, accomplished, confident, and energized… let this feeling build …. breathe in this feeling, and exhale any fears and hesitations you have…

(transition) Stay and experience this satisfaction for a few more moments. Carry this feeling and vision with you and use it to motivate you today.

Ending

Now…bring your attention back to the present. Awaken your muscles so you can continue on your journey and ready them for activity…

Episodic Future Thinking and Positive Affect and Reappraisal Condition.

Beginning

Begin by getting into a comfortable position

First, take a deep breath in and… exhale fully … continue breathing deeply, releasing any tension in your body as you exhale…. Next, take a slow breath and focus on how your body feels as you breathe…

notice the natural rhythm of your breath, and continue breathing evenly in this way…. (transition)

Prompt Future thinking/Future self

Now, take a moment to create a picture in your mind of a future ideal version of yourself—a future version of yourself who is healthy, strong and embodies what you strive for... picture in your mind a healthy body that is radiating positivity. This body has strong muscles and a vital heartbeat. It feels energized and positive.

Take a moment to think about this future version of yourself that is healthier and fulfilled.

Cue convo w/ experimenter and begin thinking of journey to future, ideal self and bring in positive affect and reappraisal

Now, as you picture this ideal version of yourself… imagine the journey you take toward your future self… imagine that on this journey you are being physically active…moving your body…accomplishing goals, and becoming stronger, more focused, and healthier…

Imagine yourself being physically active and building energy within your body. Select a physical activity to do today and imagine yourself doing this physical activity. Think of how doing this physical activity will expand your physical capabilities. Think about the positive benefits of this activity for you… how it might enable you to do more than you could before, and make your daily life feel more enjoyable…

Build on this and take a moment to imagine yourself doing this activity as vividly as you can… imagine your body moving…

What sensations are you feeling as your body moves? Is your heart beating faster, harder?…Do you feel your muscles contracting?…. as your effort increases, your breathing and physical sensations may intensify. Your body might feel warm…you might feel fatigued…you may want to label this experience as bad, or uncomfortable—recognize that this is an arbitrary label.

…Rather, the increase in sensations can be a good thing… your body is responding to the challenge you are giving it and becoming stronger, healthier, energized…

Attach no evaluations to your physical activity journey. Through your effort you are increasing your strength and physical capabilities, making the next time you exercise easier, and more familiar….

Future goal thinking

Now, take a moment to think about the physical activity goals you are striving for.

Think about how the steps you take today are getting you closer to accomplishing your goals. Imagine that you've finished your activity and you feel satisfied, accomplished, confident, and energized… let this feeling build …. breathe in this feeling, and exhale any fears and hesitations you have… …Today is a part of this journey to becoming this future, ideal version of yourself.

Now, 6 months have passed on your journey. Imagine that you have accomplished one of your physical activity goals.

Where might you be? .. … How do you feel? …Picture who might be around you...

…Perhaps you are doing activities now that you could not before… perhaps these activities take less effort now that you are more active. Stay and experience this satisfaction for a few more moments.

try to stay with this image of your future self in 6 months … Hold it in your mind. Carry this feeling and vision with you and use it to motivate you today.

It's ok if your mind wanders just return to this vision of yourself, as you carry it with you to today…. *(transition)*

Ending

Now…bring your attention back to the present. Awaken your muscles so you can continue on your journey and ready them for activity…

Episodic Recent Thinking Control Condition.

Beginning

Begin by getting into a comfortable position

First, take a deep breath in and exhale fully … continue breathing, releasing any tension in your body as you exhale…. Next, take a slow breath and focus on how your body feels as you breathe…

notice the natural rhythm of your breath, and continue breathing evenly in this way … (transition)

Recall of recent event (cues convo w/ experimenter)

Now, gently direct your focus to a moment that you remember from last week… imagine a moment where you were doing a simple activity, an activity that you do every day and that does not require much effort ….… think about the routine activity you selected. What activity are you doing?

Recognize how this simple activity is a part of your day and the purpose it has.

Imagine event….

Once you have this activity in mind… hold on to whatever image you have of the moment…imagine that you are replaying this memory of this activity in your mind from start to finish……

imagine the details as vividly as you can…

what are you doing? …..what is around you?..... how does your body feel?..... What position is your body in?…. what movements are you making?

if your mind wanders, it's ok…just gently bring your focus back to the memory… perhaps the sense of simplicity of this activity brings a sense of normalcy, or predictability…

inhale and hold briefly with the image of the activity in your mind… exhale and bring your focus back to your body… follow the breath in your body and bring awareness to the moment, to what's around you

Ending

Now…bring your attention back to the present. Awaken your muscles so you can continue on your journey and ready them for activity…
